# Confining *Trypanosoma brucei* in emulsion droplets reveals population variabilities in division rates and improves *in vitro* cultivation

**DOI:** 10.1038/s41598-021-97356-7

**Published:** 2021-09-14

**Authors:** Simone H. Oldenburg, Lionel Buisson, Thomas Beneyton, Deniz Pekin, Magali Thonnus, Frédéric Bringaud, Loïc Rivière, Jean-Christophe Baret

**Affiliations:** 1 Université de Bordeaux, Centre National de la Recherche Scientifique, Centre de Recherche Paul Pascal, Unité Mixte de Recherche 5031, 33600 Pessac, France; 2 Université de Bordeaux, Centre National de la Recherche Scientifique, Microbiologie Fondamentale et Pathogénicité, Unité Mixte de Recherche 5234, 33076 Bordeaux, France; 3 Institut Universitaire de France, 75231 Paris, France

**Keywords:** Lab-on-a-chip, Parasite biology

## Abstract

Trypanosome parasites are infecting mammals in Sub-Saharan Africa and are transmitted between hosts through bites of the tsetse fly. The transmission from the insect vector to the mammal host causes a number of metabolic and physiological changes. A fraction of the population continuously adapt to the immune system of the host, indicating heterogeneity at the population level. Yet, the cell to cell variability in populations is mostly unknown. We develop here an analytical method for quantitative measurements at the single cell level based on encapsulation and cultivation of single-cell *Trypanosoma brucei* in emulsion droplets. We first show that mammalian stage trypanosomes survive for several hours to days in droplets, with an influence of droplet size on both survival and growth. We unravel various growth patterns within a population and find that droplet cultivation of trypanosomes results in 10-fold higher cell densities of the highest dividing cell variants compared to standard cultivation techniques. Some variants reach final cell titers in droplets closer to what is observed in nature than standard culture, of practical interest for cell production. Droplet microfluidics is therefore a promising tool for trypanosome cultivation and analysis with further potential for high-throughput single cell trypanosome analysis.

## Introduction

Trypanosomes are pathogenic protozoan parasites infecting mammals in Sub-Saharan African countries. *T.b. rhodesiense* and *T.b. gambiense* sspp. are the causative agents of Human African Trypanosomiasis (HAT) better known as Sleeping sickness^1^, while *Trypanosoma congolense*, *T. vivax* and the *T.b. brucei* ssp. are the infectious agents in animal hosts leading to Animal African Trypanosomiasis (AAT) also known as Nagana^2,3^. Current treatments are complex and causing a variety of undesirable side effects, while *T.b.* infections being lethal if not treated. An extensive surveillance is required to decrease HAT cases^4,5^ which all adds up to large economic consequences in the affected areas^6,7^.

It is well known that trypanosomes are transmitted between hosts through bites of the tsetse fly (*Glossina* spp.). The repetitive switch between the insect vector and the mammal host causes a number of physiological and metabolic changes in the life cycle of the parasites^8^ such as cell cycle arrest, preparing the parasites to enter the insect vector^9^. Within the host, a well described mechanism is sudden changes of variant surface glycoproteins (VSGs) allowing adaptation to the immune system of the host. This action, known as antigenic variation, allows a small part of the population to continuously re-adapt to new immune system responses^10,11^. Both aspects require an heterogeneous behavior within the population and highlight the importance of variability in a cell population of trypanosomes. There is therefore a need for analytical methods that provide quantitative measurements of the cell response with a single cell resolution to identify variants potentially responsible for fast adaptation of the population to external changes. Microfluidics provide the capabilities to multiplex biochemical assays at high-throughput down to the single cell^12–17^. Previous studies have used microfluidic tools to study the *Trypanosoma* parasite. Using optical tweezers, single-cell trapping of trypanosomes coupled to the creation of chemical gradients, provided means to analyse the effect of drugs on the parasite^18,19^. More recently, Voyton *et al.* (2019) designed a trapping system to immobilize cells for single cell- imaging^20^. While promising, these techniques isolate single parasites from the total population where the relative low throughput of the analysis does not necessarily provide measurements representative of the variability observable in a large population. Droplet-based microfluidics enable compartmentalization of single cells allowing to study heterogeneity in a given group^21–23^, to quantitatively assess the effect of drugs at the single-cell level^13^ and to possibly isolate extraordinary variants^24,25^. It has shown to be relevant as culture platforms for cells and cell populations of bacteria^26–28^, yeast^29,30^, and mammalian cells^23^.

Here we describe a droplet-based microfluidic approach for encapsulation and cultivation of single *T.b. brucei* parasites in the drops of a water-in-oil emulsion. The droplets of controlled sizes serve as microreactors for the growth of parasites from single cells. We demonstrate the viability and the proliferation of the cells within the compartments over days. Interestingly, the variability of the division shows that a large population indeed contains highly dividing variants which would rapidly dominate the population in a bulk culture and possibly be responsible for drifts in culture. We also show that the final titer of the parasite in droplets exceed the maximal titer in standard cultures. Emulsion cultivation therefore appears to be a powerful cultivation method for trypanosomes in droplets leading to titer closer to the titers found *in vivo*, without the need for complicating infrastructure or animal vectors.

## Results

### Drop encapsulation of *T.b. brucei*

Bloodstream forms of *T.b. brucei* are cultivated in 24-microwell plates. All survival and growth experiments are initiated from the same starting culture to reduce population variation between different stock cultures. Growth in standard bulk culture is monitored as a control at the beginning and the end of the experiments, to ensure that the observed differences are not caused by unstable growth from an aging or drifting population (Fig. S1).

**Figure 1:**
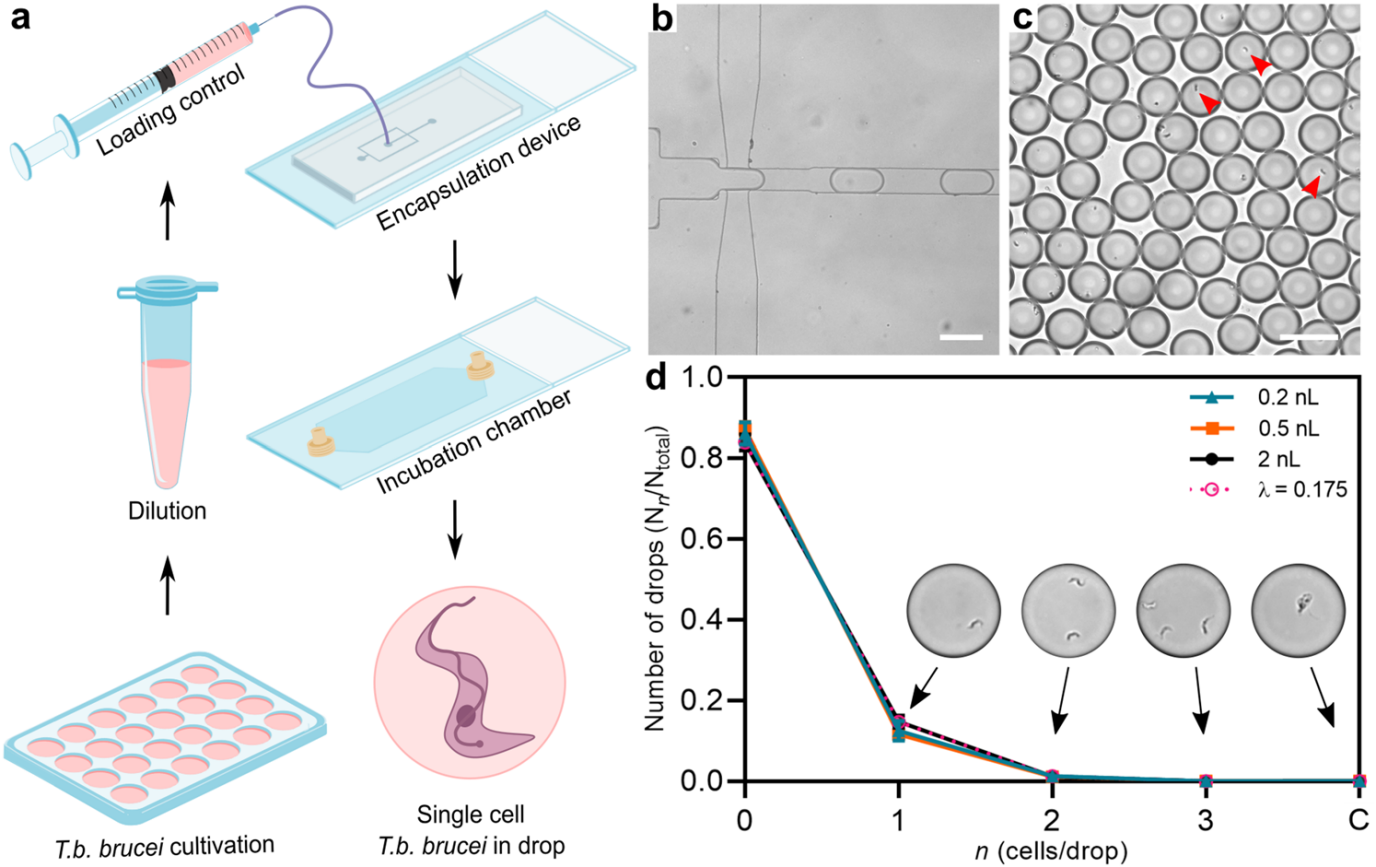
Single cell encapsulation of *T.b. brucei*. (**a**) Overview of experimental procedure. Starting from a classical cell culture, *T.b. brucei* cells are diluted to match the respective drop sizes used. After, the cell suspensions are loaded into syringes enabling connection to microfluidic devices. From encapsulation, the drops are loaded into an incubation chamber where visualization of encapsulated *T.b. brucei* takes place. (**b**) Microscopic image of drop-making process in personalized encapsulation device visualized through $\times$10 microscopic objective (Fig. S2a). (**c**) Fixed droplets containing encapsulated cells displayed in an incubation chamber visualized through $\times$10 microscopic objective. Red arrows highlight examples of encapsulated cells. (**d**) Poisson distribution of three different sizes of drops together with the predicted statistic values with a cell distribution of 0.175 cells per drop. All data are presented as mean values ±SD made as biological replicates (n = 3). Inserted images show examples of drops containing one, two, or three cells and an uncountable quantity defined as ‘clumps’ (C) using a $\times$20 microscopic objective. All scale bars are set to 100 $\mu$m. Images displayed in **a** are used directly or modified in Inkscape (https://inkscape.org) from SMART (Servier Medical Art), licensed under a Creative Common Attribution 3.0 Unported License (https://smart.servier.com).

We use two devices to respectively encapsulate and incubate single cell *T.b. brucei* and quantify their growth in nL volume microcompartments (Fig. S2). Cell suspensions are loaded into syringes in prior to injection in the microfluidic devices (Fig.1a). For drop production, we use a dual flow device with fluorinated oil containing surfactant pinching the cell suspension flow creating equal sized microcompartments in the form of drops (Fig. 1b, Fig. S2a). The flow rates of the oil and cell suspension together with the dimension of the nozzle in the respective devices are controlling the drop sizes to reach final drop volumes $V_{drops}$ of 0.2 nL ±1.80$\%$, 0.5 nL ±1.84$\%$ and 2 nL ±2.61$\%$. For incubation, the drops are collected in an incubation chamber stabilizing the drops in a monolayer display allowing the exact same drops to be analysed over periods of time, as described previously (Fig. 1c)^25^. The incubation chamber is connected to the outlet of the encapsulation device by external tubings and has a volume of approximately 18 $\mu$L (Fig. S2b). One incubation chamber therefore provides multiplex measurement of over 10$^4$ droplets. After filling, the incubation chamber is detached from the encapsulation device and completely sealed enabling the transfer of the chamber to a cell incubator or a microscope when needed.

In order to investigate cell survival upon isolation, we optimized the number of drops containing only single cells, while keeping the number of drops containing more than one cell at a minimum, by dilution of the encapsulated cell suspensions (in the range $C = 0.875-8.750\cdot 10^{5}$ cells/mL). The cells being randomly distributed in the bulk suspension, the cell distribution is expected to follow a Poisson distribution: f($\lambda ;n)=\lambda ^ne^{-\lambda }/{n!}$, where *n* is the actual number of cells in the drops and $\lambda$ is the average number of cells per drop^23^. $\lambda$ relates directly to the cell density *C* in the culture as $C = \lambda / V_{drops}$. Using an occupation factor $\lambda$ = 0.175, we recover cell distributions consistent with a Poisson distribution for the three sizes of drops (Fig. 1d). We obtain $\sim$ 11–15% drops containing single cells in drop sizes of 0.2 nL, 0.5 nL and 2 nL. For all three sizes, less than 1.5% of drops contain more than one cell. In few of the drops we observe a cluster of several cells clumping together (Fig. 1d), referred to as clumps (C). This is a common event in *in vitro* cultures when populations are reaching high cell densities. Except for these rare events, the encapsulation of trypanosomes is reliable and equivalent to the encapsulation of any type of particles randomly distributed in the initial volume.

### Survival and growth of *T.b. brucei* in drops

**Figure 2:**
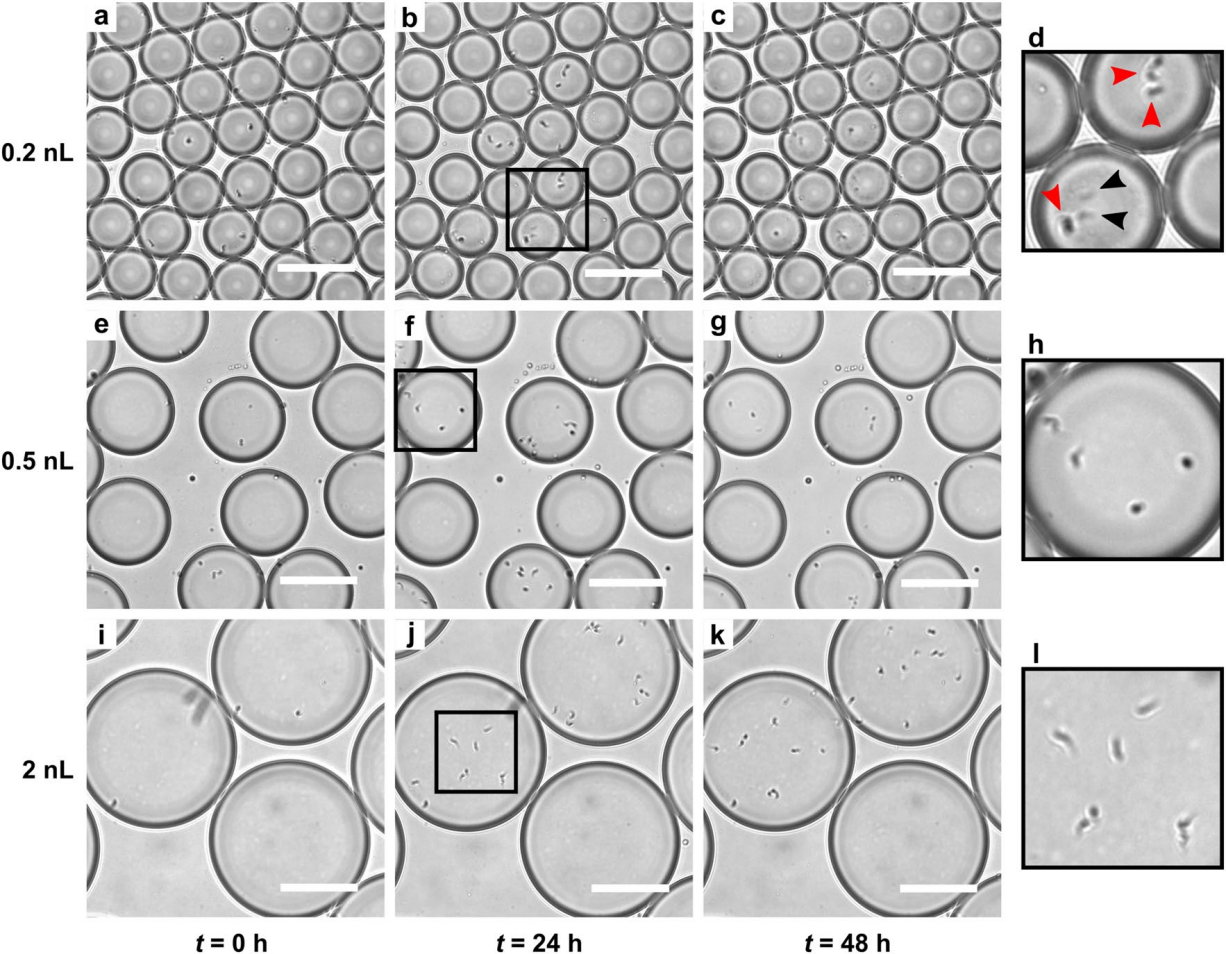
Impact of drop size on trypanosome survival. The figure display snapshots of encapsulated *T.b. brucei* cells just after encapsulation, after 24 h of incubation and after 48 h of incubation in drop sizes of 0.2 nL (**a–c**), 0.5 nL (**e–g**) and 2 nL (**i–k**), visualized in incubation chamber through a x10 microscopic objective. Examples of living cells (red arrows) and dead cells (black arrows) are seen in the close-up snapshot (**d**) at 24 h. In the snapshots (**h**, **l**) all cells are alive. All scale bars are 100$\mu$m. For snapshots **a–c**, **e–g** and **i–k**, the corresponding original movies, showing survival based on motion of the trypanosomes, are provided as supplementary (Movie S1–S9).

We first determine the survival of cells in droplets as a function of droplet sizes (Fig. 2, supporting Movie S1–S6). Droplets containing cells are collected in the incubation chamber in which 200–400 cell-containing drops are counted. All counting experiments are performed in triplicates for each experimental condition. The cells are counted directly from the incubation chamber by eye every 4 h during daytime until death occurs and the experiments are repeated for each drop size (Fig. 2a–k). Cell death is determined when no movement is observed in a time period of 2–3 min. In the micrographs, a dead cell is identified as cell debris appearing as fainted traces (black arrows Fig. 2d).

**Figure 3:**
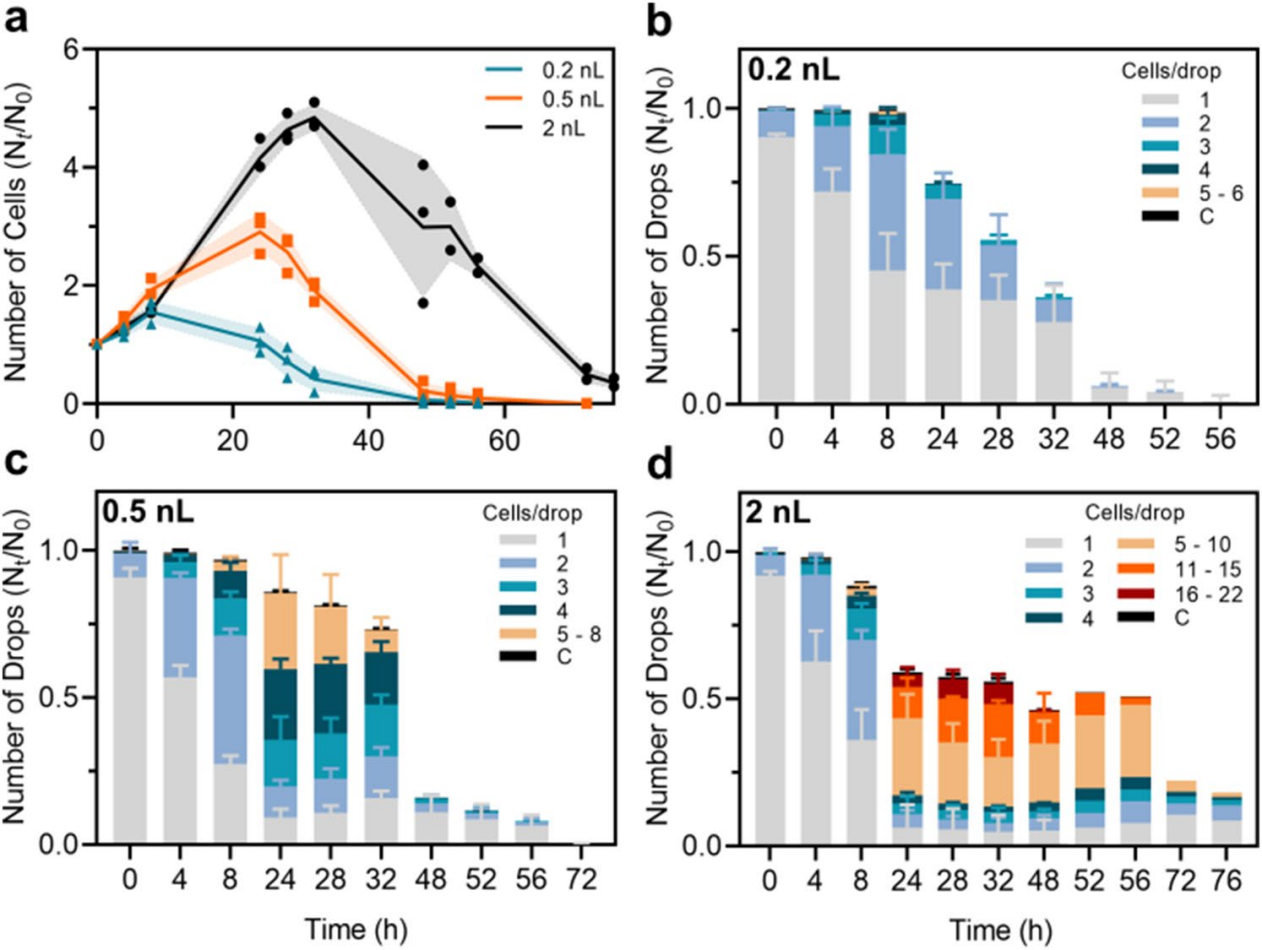
Survival and growth of encapsulated *T.b. brucei*. (**a**) Comparison of cell survival, defined as normalized total cell number, between droplet sizes of 0.2 nL (blue), 0.5 nL (orange) and 2 nL (black) over time. Supporting the cell survival data is the presentations of growth over time defined as normalized number of drops containing a given number of cells in 0.2nL drops (**b**), 0.5 nL drops (**c**) and 2nL drops (**d**). All results are presented as mean values ±SD. Experimental data from 0.2 nL, 0.5 nL and 2 nL drops (0–48 h) are made as biological replicates (n = 3). For 2 nL drops at time 52 to 76 h n = 2. Significance values supporting the survival differences observed in (**a**) are presented in Table 1, 2 and 3.

We analyze the influence of the drop size on survival and growth of encapsulated *T.b. brucei* (Fig. 3). In the 0.2 nL drops, the total cell number increases by a factor of 1.5 during the first 8 h of incubation to reach a maximum. After 24 h, the total cell number has reached down to the initial level and further decreased until all cells have died (after 56 h, Fig. 3a,b). In the 0.5 nL drops, the total cell number increases by a factor of 3 with a maximum at 24 h. All cells are dead after 72 h (Fig. 3a,c). Finally, in the 2 nL drops, the total cell number has increased by a factor of 5 at the maximum after 32 h of incubation. After 76 h when the experiment is terminated, a small fraction of cells remains alive (50% of the initial count, Fig. 3a,d). The initial growth dynamics is similar for all volumes over the first 8 h of incubation. For larger incubation times the growth dynamics strongly depends on the droplet size. From 8 to 32 h of incubation we observe a significant difference in growth between all the drop sizes, with bigger drops reaching a higher total cell number (Table 1, 2 and 3).

**Table 1:** Difference in cell survival between 2 nL and 0.5 nL drops. Statistical differences are presented as* P* values. Two-sided Student’s* t* test was used to find significance values. Effect sizes were calculated as Hedges’ *g*^57^.* P* > 0.05 = non significant (ns),* P*$\,\le\,$0.05 = *,* P*$\,\le\,$0.01 = **,* P*$\,\le\,$0.001 = *** and* P*$\,\le \,$0.0001 = ****.

Time (h)	*P* value	Significance	Effect size (*g*)
4	0.34665	ns	0.87
8	0.01795	*	3.16
24	0.00741	**	4.09
28	0.00083	***	7.39
32	5.25$\cdot$10$^{-5}$	****	14.94
48	0.01597	*	3.28
52	0.00283	**	8.28
56	0.00024	***	19.17
72	0.00658	**	6.18
76	–	–	–

**Table 2:** Difference in cell survival between 2 nL and 0.2 nL drops. Statistical differences are presented as* P* values. Two-sided Student’s* t* test was used to find significance values. Effect sizes were calculated as Hedges’ *g*^57^.* P* > 0.05 = non significant (ns),* P*$\,\le\,$0.05 = *,* P*$,\le\,$0.01 = **,* P*$\,\le\,$0.001 = *** and* P*$\,\le\,$0.0001 = ****.

Time (h)	*P* value	Significance	Effect size (*g*)
4	0.38852	ns	0.79
8	0.84538	ns	0.17
24	0.00011	***	12.36
28	4.26$\cdot$10$^{-5}$	****	15.75
32	1.34$\cdot$10$^{-5}$	****	21.05
48	0.01305	*	3.48
52	0.00233	**	8.85
56	–	–	–
72	–	–	–
76	–	–	–

**Table 3:** Difference in cell survival between 0.5 nL and 0.2 nL drops. Statistical differences are presented as* P* values. Two-sided Student’s * t* test was used to find significance values. Effect sizes were calculated as Hedges’ *g*^57^.* P* > 0.05 = non significant (ns),* P*$\,\le\,$0.05 = *,* P*$\,\le\,$0.01 = **,* P*$\,\le\,$0.001 = *** and* P*$\,\le\,$0.0001 = ****.

Time (h)	*P* value	Significance	Effect size (*g*)
4	0.07137	ns	2.00
8	0.05461	ns	2.20
24	0.00126	**	6.62
28	0.00144	**	6.39
32	0.00057	***	8.12
48	0.15578	ns	1.43
52	0.20194	ns	1.25
56	–	–	–
72	–	–	–

We have shown that single cell *T.b. brucei* is indeed able to survive and grow upon isolation in a time span of hours to days, with longer survival and higher cell number per droplet measured in larger droplets.

### Growth variability between drops

**Figure 4:**
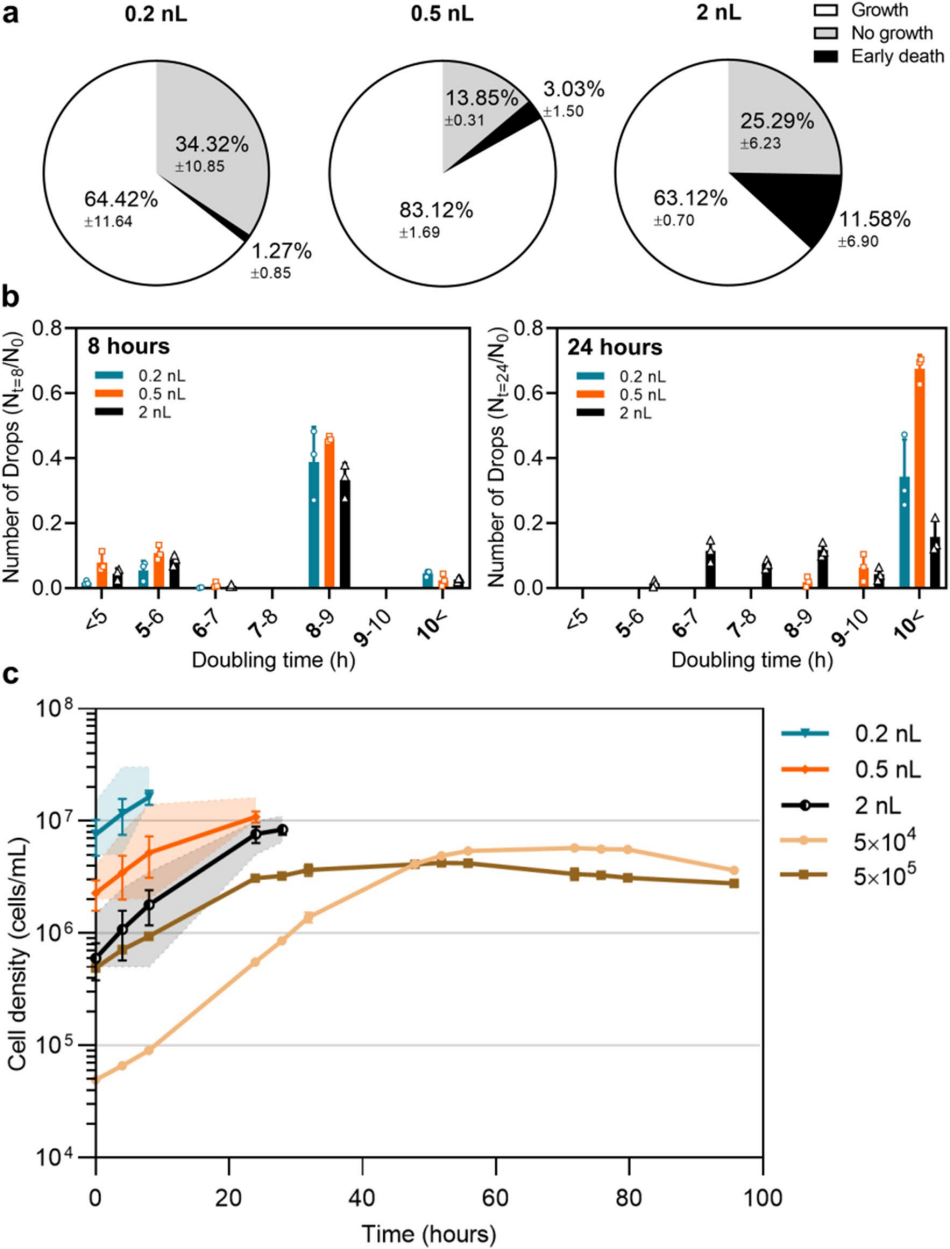
Growth variabilities within drop populations. (**a**) Divisions of the total populations in three main behaviors; normal growth (white), survival for more than 8 h without division (grey) and early death occurring before 8 h (black) in 0.2 nL, 0.5 nL and 2 nL drops. (**b**) Comparison of doubling times at 8 h and 24 h between 0.2nL drops (blue), 0.5nL drops (orange) and 2 nL drops (black). Doubling times represented by bold values are included in the respective group. (**c**) Comparison of growth curves between drop cultivation (blue, orange and black lines) and standard cultivation (brown lines) techniques. Droplet growth curves represent the fastest growing populations in 0.2 nL, 0.5 nL and 2 nL droplets. The shaded areas show the lower and upper limits of the cell numbers. Standard growth curves are initiated from respectively 5$\cdot$10$^{4}$ and 5$\cdot$10$^{5}$ cells/mL. Results in (**a**) and (**b**) are presented as mean values of biological triplicates ±SD. Results in (**c**) are presented as mean values ±SD of biological or technical triplicates for respectively drop- and standard cultivation curves.

We have focused on the survival and growth between different sizes of drops. A further analysis of the data reveals three general types of growth behaviours regardless of drop size (Fig. 4a). By tracking the individual drops over time, we observe a minor part of the drop populations containing cells dying within the first 8 h of encapsulation. In 2 nL drops, early death is observed in 11.58% of the drop population, where in 0.2 nL and 0.5 nL drops early death is seen in 1.27% and 3.03% of the populations, respectively. In a huge fraction of the drop populations, surviving cells do not undergo cell division at any time during their lifetime occurring in 14% to 34% of the cases (Fig. 4a). The majority of the drop populations contain cells undergoing minimum one cycle of cell division with doubling times varying from > 10 h to < 5.5 h after 8 and 24 h of incubation (Fig. 4a,b). At 8 h, all drop sizes have similar growth where 40–50% of the populations contain cells with doubling times in the range of 8 h, and 10–20% of the drop populations contain cells with doubling times in the range of 5 h or less (Fig. 4b (left)). At 24 h of incubation, the remaining living cells of 0.2 nL drops and the majority of the 0.5 nL drops have doubling times higher than 10 h. The doubling times in the 2 nL drops are about evenly distributed, in the range from 6 h up to 10 h and higher (Fig. 4b (right)). In the fast growing drops, the maximum cell densities reached are 22, 8 and 6 cells per drop in respectively 2 nL, 0.5 nL and 0.2 nL drops. In order to compare the droplet conditions, we calculate the cell volume density. The maximum cell density in the 2 nL drops reaches a maximum of 8–11 cells/nL, in 0.5 nL drops 10–16 cells/nL and in 0.2 nL drops reaching a maximum of 15–30 cells/nL (Table 4).

**Table 4:** Maximum cell numbers reached in droplet cultivation. Overview of maximum cell number (cells per drop) reached in the three drop sizes and the respective cell volume densities (cells per volume).

Droplet size (nL)	Max. cell number per drop	Max. cell number per nL
0.2	3–6	15–30
0.5	5–8	10–16
2	16–22	8–11

We plot growth curves corresponding to the growth observed in the drops reaching the maximum cell densities and compare with growth from standard in bulk techniques (Fig. 4c). In the standard growth curves two different starting concentrations are used and cells are counted every 4 h during daytime until cell densities start decreasing. In classic growth curves the maximum cell densities reach 4–5$\cdot$10$^{6}$ cells/mL, with the lowest starting concentration reaching the highest density. The average of the maximum cell densities reached in drop cultivation is 2$\cdot$10$^{7}$ cells/mL in 0.2 nL drops, 1$\cdot$10$^{7}$ cells/mL in 0.5 nL drops and 8$\cdot$10$^{6}$ cells/mL in 2 nl drops.

We have here measured a large variability in division within populations of *T.b. brucei* and shown higher maximum cell densities in droplet cultivation over standard in bulk techniques for the highly dividing fraction of cells.

### Up-scaling of drop yield

**Figure 5:**
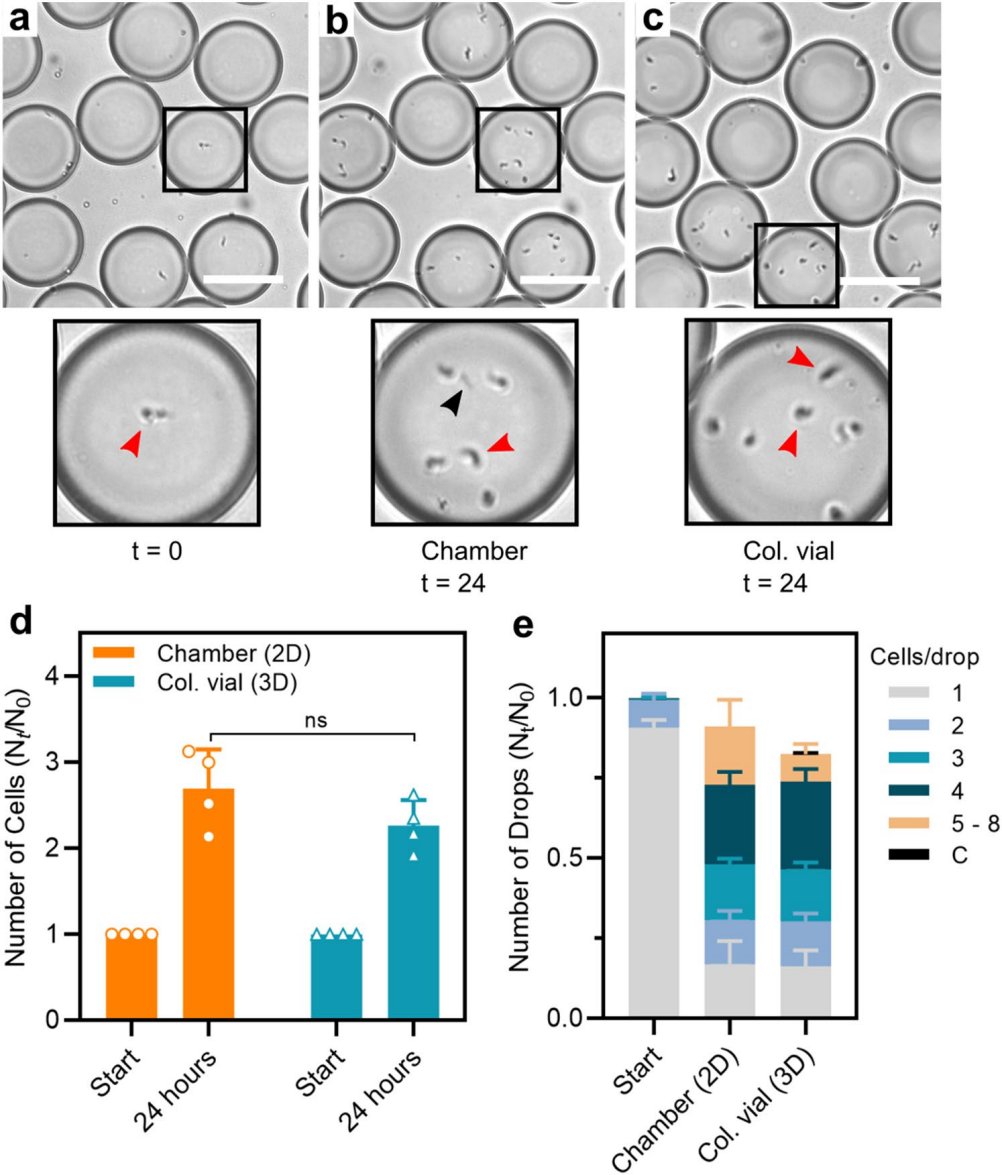
Collection and incubation of 0.5 nL drops in chamber vs. collection vial. (**a**) Snapshots of encapsulated *T.b. brucei* cells at beginning of incubation, (**b**) after 24 h of incubation in chamber and (**c**) after 24 h of incubation in collection vial, visualized through a x10 microscopic objective. Micrographs display close-up examples of encapsulated trypanosomes in drops of the respective collection devices. (**d**) Comparison of cell survival as normalized values after collection and 24 h of incubation in incubation chamber and collection vial. (**e**) Supporting cell survival data is the presentations of growth after 24 h as normalized number of drops containing a given number of cells in the different collection devices. All data are presented as mean values ±SD made as biological replicates (n = 4). Two-sided Student’s * t* test were used to find significance value (*P* = 0.16) in (**d**).

After establishing encapsulation and cultivation of single-cell trypanosomes in microcompartment droplets, we analyse the potential of up-scaling the final drop yield (Fig. 5). *T.b. brucei* are encapsulated in 0.5 nL drops and collected for 15 min. off-chip in a 200 $\mu$L plastic vial providing a collection of > 10$^{5}$ droplets, allowing multiple usage of the drops by re-injection from the collection vial. After collection, the inlet tubing is detached and closed and the vial is moved to incubation. For control, drops are simultaneously collected in the incubation chamber, where an estimate of 300 cell containing drops are counted, functioning as time zero for both collection devices (Fig. 5a). After 24 h of incubation the cell-containing drops are counted in the incubation chamber (Fig. 5b) where drops from the collection vial subsequently are re-injected and counted (Fig. 5c).

We analyse how the survival and growth of *T.b. brucei* in drops are influenced by collection and incubation in the larger collection device. After 24 h of incubation the total cell number increases by a factor around 2.5 in both collection devices (Fig. 5d). A small non-significant increase in total cell number is seen in the incubation chamber compared to the collection vial (*P* = 0.16). The cell densities inside the individual drops are likewise similar between growth in the incubation chamber and the collection vial (Fig. 5e). In comparison with the previous survival and growth results of encapsulated *T.b. brucei* in 0.5 nL drops, we furthermore obtain almost identical results between the two experiments emphasizing good experimental repetitiveness (Fig. S3).

We have shown that drop production can easily be up-scaled to achieve a higher drop yield without influencing survival and growth of the trypanosomes. We also emphasize the reproducibility of the survival and growth assays executed with several months in between.

## Discussion

We have here developed a droplet-based microfluidic system for creation of microcompartment droplets to successfully encapsulate and cultivate the *T.b. brucei* parasite on a single-cell level (Fig. 1, Fig. 2). We have shown how encapsulated trypanosomes survive in micron-sized droplets from several hours to days (Fig. 3). The droplet sizes are found to positively correlate with the survival time of the encapsulated cells, making larger droplets preferred for long-term experiments.

During trypanosome infection the parasitemia level of the infected mammal is varying from 10$^{7}$ cells/mL up to 10$^{9}$ cells/mL with large fluctuations during the time period of the infection^31–33^. However, in *in vitro* cultivation the obtained titters of the *T.b. brucei* bloodstream forms are much lower. By standard laboratory techniques cell densities reach a maximum of 4–5$\cdot$10$^{6}$ cells/mL^34–36^. Different techniques involving continuously medium refreshment have previously been described to improve the poor yield of *in vitro* trypanosome cultivation^35,37^. However, by cultivating trypanosomes in drops we here reach cell densities 10-fold higher than by standard cultivation without the need of medium or nutrition replacement. Interestingly, while bigger drops have the longest survival, we observed that smaller drops lead to a higher cell density. With a maximum density of 1.1$\cdot$10$^{7}$ cells/mL in 2 nL drops, 0.2 nL drops reached a max density 3 times higher with 3$\cdot$10$^{7}$ cells/mL (Fig. 4, Table 4). A similar correlation has previously been observed, where survival time of encapsulated human cells showed an inverse correlation with the cell density^23^. Worth noting, by simply increasing the starting concentration in standard cultivation techniques we did not observe a higher final cell density (Fig. 4). Thus, cultivation in droplets seem to benefit the trypanosome parasite, not because of a higher starting cell density but possibly due to the small confined volumes.

By cultivating single *T.b. brucei* cells in drops, we show that droplets of the same size do not promote identical growth (Fig. 4). Where in standard cell-based research, we assume that average activities from a population represent the typical cell, we here reveal various cellular growth patterns within a population. Worth emphasizing is the existence of various growth patterns in standard culture condition as well as in droplet cultures. However, in standard conditions fast growing cells are likely to quickly dominate a population and hide the slower growth rates that we here reveal in drops. While some cells reach doubling times as seen in bulk, most cells exhibit slower or none growth ranging from around 6–24 h after droplet encapsulation. Those cells would be out-competed in a bulk experiment showing how a small fraction of fast dividing cells might quickly become dominant in the bulk culture and a possible reason of adaptation in the population. Heterogeneity in monoclonal populations is a well known phenomena derived from events as stochastic switching or noise present in both prokaryotic and eukaryotic cells^38–40^. In the protozoan parasites *Leishmania* and *Trypanosoma cruzi*, belonging to the same taxonomic family as *T.b. brucei*, cell to cell variation has been shown by stochastic DNA amplification and clonal variation in stress-related responses^41,42^. Variabilities within *T.b. brucei* populations have already been shown to occur during adaptation from the insect vector to the mammal host and back again^8,10,11^. These transitions, causing a number of physiological and metabolic changes, indicate a heterogeneous behaviour inside the populations. Single-cell analytical methods like microfluidics are therefor highly desirable to further study these heterogeneous behaviors and to discover new variabilities or behaviors. Although other studies have already coupled microfluidic approaches to study trypanosomes on a single-cell level^18–20^, no successful attempts have been reported on isolating a single trypanosome in enclosed compartments. In this work, droplet-based microfluidics show promising for single-cell compartmentalisation and cultivation of the trypanosome parasite. By moving from a two-dimensional collection chamber to a three-dimensional collection vial, we maintained the same survival and growth patterns in an environment allowing continual usage of the collected drops by reinjection (Fig. 5).

A next step to investigate population variability is a further analysis of the highest dividing variants. Coupling our droplet-cultivation approach with additional sorting modules will allow us to select droplets containing the most dividing cell variants. Such systems have previously been shown to successfully screen and select drops based on content densities^43–45^. Other well described approaches are using fluorescence-based sorting for high-throughput analysis of cellular activities on a single cell level^23,46–48^. Using fluorescence-based assays is another promising tool to study population variability by analysing enzymatic activities between individual cells. Finally, obtaining genetic information of variants with prominent division rate or enzymatic activity in populations of trypanosomes is of great interest. Droplet-based sequencing allow the acquisition of genetic profiling of individual cells^49–51^ and can possibly lead to the identification of new drug targets against the disease.

## Conclusion

We have here established a system for encapsulation and growth of *T.b. brucei* in microcompartment droplets at the single-cell level. We have shown that trypanosomes survive from several hours to days being sufficient time for several cycles of cell division. We see that differences in droplet size influence the survival and division: qualitatively, cells survive longest in the largest drops (2 nL) reaching higher total cell numbers per drop, but simultaneously the highest cell densities (cells per volume) is reached in the smallest droplets (0.2 nL). We observed an heterogeneous pattern of growth behavior between the individual cells within a population, indicating that some cell variants divide more efficiently while others divide slower than the average. Cultivating trypanosomes in droplets also resulted in 10-fold higher cell densities among the fastest dividing variants compared to standard cultivation techniques. This improvement is achieved in drops without the need of additional maintenance methods and is potentially interesting to optimize cultivation techniques of the parasite. Finally, these effects quantitatively characterized in two-dimensional microfluidic systems is shown robust. The same improvement in cell densities is obtained for incubation of the cells in a three-dimensional macroscopic emulsion.

Our results demonstrate the interest to cultivate single cell trypanosomes in microcompartment droplets, and represent a future opportunity to develop a droplet-based microfluidic platform to investigate behaviors and activities on a single cell level. Further adaptation of our system can lead to numerous applications as droplet sorting and screening to advance the exploration of new drug candidates against the trypanosome parasite.

## Methods

### Cell preparation

Cells are cultivated using standard cultivation protocols^34^. In brief, monomorphic *Trypanosoma brucei brucei* 427 90-13 bloodstream form (BF) were grown in Iscove’s Modified Dulbecco’s Medium (IMDM) (Life Technologies) supplemented with 10% (v/v) heat-inactivated fetal calf serum (FCS), 0.2 mM $\beta$-mercaptoethanol, ﻿36 mM NaHCO_3_, 1 mM hypoxanthine, 0.16 mM thymidine, 1 mM sodium pyruvate, 0.05 mM bathocuprone, 1.5 mM L-cysteine, $40 \mu$g $\cdot$ mL$^{-1}$ streptomycin and 40U $\cdot$ mL$^{-1}$ penicillin. *T.b. brucei* BF were maintained as one-mL cultures in 24-well Nunc plates (Thermo Fischer Scientific) at $37^{\circ }$ C with 5% $\hbox {CO}_{2}$. Every 2 or 3 days cultures were sub-cultured with a 1.000 or 10.000 fold dilution, respectively. In prior to all encapsulation steps, cells are counted in a Guava easyCyte Flow cytometer (EMD Millipore). All doubling times, $d_t$, for bulk and droplet cultivation are calculated as $d_t = (t_2 - t_1) \cdot (log(2)/log (\frac{q_2}{q_1}))$, where $t_1$ and $t_2$ is the time of the beginning and the end of the time span for the wanted doubling times, and $q_1$ and $q_2$ are the start and the end cell numbers for the respective time span.

### Device design and fabrication

Microfluidic encapsulation devices were made of poly-(dimethylsiloxane) (PDMS, Sylgard 184) from SU8-3000 negative photoresists (MicroChem Corp) molds produced using standard soft lithography techniques^52^. Briefly, negative photo resist SU-8 3050 or SU-8 3025 were spin-coated on silicon wafers (Brand) and exposed to UV light through customized patterned transparent photomasks (CAD/Art Services, Inc., SELBA S. A. for high-resolution printing (50800 dpi)) using a MJB4 mask aligner (SUSS MicroTec). After UV exposure the molds are developed by SU-8 developer (MicroChem). A mixture of PDMS/curing agent (10% w/w, Sylgard 184 kit) was poured on the molds, degassed in a vacuum chamber and cured at 70$^{\circ }$C for at least 2 h. The PDMS was peeled from the molds and inlet and outlet holes were punched with a 0.75mm-diameter puncher. Prepared PDMS slabs and microscope glass slides were cleaned, dried and exposed to oxygen-plasma treatment (Pico Low pressure plasma system, Diener electronic) in prior to bonding by pressing the two components together. The surface of the microfluidic channels were treated using fluoro-silane (Aquapel, PPG Industries) before use. All devices were flushed with argon before and after Aquapel treatment. Devices for encapsulation were fabricated in three different sizes according to the desired size of drops. For drop production with sizes of 0.2 nL, the channel depths of the devices were 30 $\mu$m and nozzle widths 50 $\mu$m. For sizes of 0.5 nL, channel depths were 85 $\mu$m and nozzle widths 50 $\mu$m. For drops of 2 nL, channel depths were 95 $\mu$m and nozzle widths 100 $\mu$m. To observe drops as a 2D-array, an incubation chamber was fabricated as previously described^25^. Glass microscopy slides were used as top and bottom covers (76$\times$25$\times$1mm). Two holes were created in the top glass slide by micro-sandblasting. Both slides were cleaned using soap, water and ethanol and dried at 70$^{\circ }$C. The desired chamber design was cut in a 60 $\mu$m thick double-sided bonding tape (1375, SDAG, Adhesif) using a Graphtech cutting plotter (CE6000-40). The two cleaned microscope slides were attached to each other using the bonding tape. 10-32 NanoPort assembled with NanoTight fittings (IDEX Health and Science) were glued on the inlet holes with UV curing glue (Loctite 3529, Henkel)(Fig. S2b). The inner dimension of the incubation chamber was appx. 1$\times$3 cm and 60 $\mu$m in depth corresponding to a volume of 18 $\mu$L. The chamber was treated with fluoro-silane (Aquapel, PPG Industies) and dried with argon prior to use. The chamber was reused multiple times and cleaned between each experiment by flushing fluorinated oil.

### Optical setup

For drop observation and size measurements, we used a homemade laser-induced epifluorescence setup, implemented on an inverted microscope (Axiovert 135, Carl Zeiss) (Fig. S4). Fluorescence was excited by a continuous laser at 473 nm (Cobolt 06-MLD, Cobolt AB) focused directly on the drops by the observation objective (Zeiss LD Achroplan 40x/0.6 Korr Ph2). Fluorescence was filtered by a band pass filter at 525 nm and 50 nm width (525/50 BrightLine HC) and collected by a photo multiplier tube (PMT) (H10723, Hamamatsu Photonics K.K.) at 10kHz with a NI acquisition card (NI USB 6008), which was controlled by a homemade LabVIEW program. Fluorescence collection was done in confocal configuration to reduce background noise. A Blackfly S USB3 camera (FLIR Systems) was mounted on the top camera port of the microscope where the ocular piece have been removed. The entire setup was fixed on an aluminium platform with vibration dampening feet to reduce vibration noise from the surroundings.

### Cell encapsulation and incubation

Cells were grown to a density of 1.2–3$\times$10$^{6}$ cells$\cdot$ mL$^{-1}$ in prior to each experiment. The cell suspensions were further diluted to a value corresponding to 0.175 cells/drop in each of the wanted drop sizes. Upon drop formation, cell suspensions were emulsified using HFE-7500 fluorinated oil (3MTM Novec) stabilized by FluoSurf surfactant (emulseo) with a final concentration of 2.5% (v/v). Cell suspension and fluorinated oil were separately loaded into 1 mL Omnifix-F syringes (B Braun) with 0.4$\times$19 mm needle tips (Terumo) and pumped into the drop making device by low pressure neMESYS syringe pumps (CETONI GmbH) with flow rates corresponding to the wanted drop sizes. The drop sizes were determined as $V_{drops} = {Q}/{f}$, where *Q* was flow rate of the cell suspension in $\mu$L/h and *f* was the drop frequency calculated from laser measurements (Fig. S4 text). For 0.2 nL drops and 0.5nL drops the flow rates of the fluorinated oil were 300 $\mu$L$\cdot$ h$^{-1}$ and 850 $\mu$L$\cdot$ h$^{-1}$ and for the cell suspension 280 ± 10 $\mu$L$\cdot$ h$^{-1}$ and 250 ± 20 $\mu$L$\cdot$ h$^{-1}$, respectively. For 2 nL drops, the flow rate of the fluorinated oil was 750 $\mu$L$\cdot$ h$^{-1}$ and 330 ± 20 $\mu$L$\cdot$ h$^{-1}$ for the cell suspension. Syringes were connected with microfluidic devices by polyethene tubing (Adtech) in dimensions of 0.3 mm inner diameter and 0.76 mm outer diameter. After drop making, the emulsion was collected off-chip in an incubation chamber or a collection vial. After collection, the emulsion-containing collection device was incubated at 37$^{\circ }$C with 5% $\hbox {CO}_{2}$ until further use. All cell counting were performed by eye directly from the incubation chamber containing the emulsified cells during a time span of 30–45 min. For all drop and cell counting upon fixation the in incubation chamber, a microscope incubator (Okolab) was used set at 37$^{\circ }$C.

### Statistical analysis

Statistical analyses were performed between all drop sizes at each time point during the survival assay (Fig. 3a) in order to find the significance values presented in Table 1, 2 and 3. We used a Student’s * t* test (parametric, unpaired, two-tailed analysis) to determine significance between the mean survival of the different drop sizes^53,54^. Supplementary we calculated the effect size for each analysis, which previously has been recommended when working with small sample sizes^55–57^.* T* tests were performed in Microsoft Excel (Microsoft Corporation) and by GraphPad prism version 8 (GraphPad Software) supporting the findings. Effect sizes were found using the online tool Effect Size Calculator for tests (https://www.socscistatistics.com/effectsize/default3.aspx). Alpha level for all analyses were 0.05.

## Supplementary Information

Supplementary Information 10.

Supplementary Information 2.

Supplementary Information 3.

Supplementary Information 4.

Supplementary Information 5.

Supplementary Information 6.

Supplementary Information 7.

Supplementary Information 8.

Supplementary Information 9.

Supplementary Information 10.

## Data Availability

All relevant data are available from the corresponding author upon request.
