# Supplementary material for: Confining Trypanosoma brucei in emulsion droplets reveals population variabilities in division rates and improves in vitro cultivation
Source: Sci Rep. 2021 Sep 14;11:18192. doi: 10.1038/s41598-021-97356-7 (PMC8440574; doi:10.1038/s41598-021-97356-7)
Supplement: Supplementary file 10 — Supplementary Information 10. [file 41598_2021_97356_MOESM10_ESM.pdf]

Confining *Trypanosoma brucei* in emulsion droplets reveals population variabilities in division rates and improves *in vitro* cultivation

Simone H. Oldenburg,[1,2] Lionel Buisson,[1] Thomas Beneyton,[1] Deniz Pekin,[1] Magali Thonnus,[2] Frédéric Bringaud,[2] Loïc Rivière,[2] and Jean-Christophe Baret[1,3]

[1] *Université de Bordeaux, Centre National de la Recherche Scientifique, Centre de Recherche Paul Pascal, Unité Mixte de Recherche 5031, Pessac, 33600, France*

[2] *Université de Bordeaux, Centre National de la Recherche Scientifique, Microbiologie Fondamentale et Pathogénicité, Unité Mixte de Recherche 5234, Bordeaux, 33076, France*

[3] *Institut Universitaire de France, Paris, 75231, France*

Supplementary text

Growth stability

To ensure that any observed variability was not caused by changed or unstable growth from age differences or population drifting, we made growth curves of the starting bulk cultures (Fig. S1). During the first and the last two weeks of the of the experimental time span, the cells were counted every day and diluted back to the initial densities where doubling times were calculated from the cumulative cell numbers.

Microfluidic devices

Two microfluidic devices were used for respectively cell encapsulation and droplet incubation (Fig. S2). Cell encapsulation devices are made from negative photoresists molds by standard soft lithography techniques [1]. The two-dimensional incubation chamber are fabricated as described in the main text [2].

Long term repetition

After testing the possibility of up-scaling the drop yield (main text Fig. 5), we compared the results between the up-scaling growth experiment with the initial survival and growth experiments (main text Fig. 3) both from incubation in the two-dimensional chamber. Up-scaling experiment are here referred to as 'New data' where the initial survival and growth experiments are referred to as 'Previous data' (Fig. S3). The Previous data and the New data were made with a eight month gab, where all material conditions were renewed. With the use of new batches of cells, culture medium and surfactant, no significant difference (P = 0.53) was observed in survival and growth between encapsulated *T.b. brucei* in 0.5 nL droplets after 24 hours of incubation.

Optical setup

All experiments were executed on a homemade laser-induced epifluorescence setup build on an inverted microscope (Fig. S4). Syringe pumps were controlling the flows of the devices which together with excited fluorescence by a continuous laser (473 nm) were used to calculate the exact droplet volumes. The laser was passed by an optical density filter (OD) and focused directly on the drops through the microscope objective directed by a mirror (M) and a dichronic mirror (DM). Fluorescent signal was directed back through the DM, a notch filter (NF) and split by a beam splitter (BS). One beam was directed to the camera, where the other was filtered by a band pass filter (BF) and collected by the PMT with a NI acquisition card (DAQ). The fluorescent signal acquisition was controlled on the computer by a LabVIEW program. To reduce background noise fluorescence collection was done in confocal configuration through a pinhole (P).

Droplet volumes

Droplet volumes were determined as $V_{drops} = Q/f$ with Q as the cell suspension flow rates in $\mu L/h$ and f as droplet frequency. The droplet frequency were calculated from the signals obtained by the laser measurements as $f = N_{drops}/\Delta t$. Background fluorescence from the cell culture medium made it possible to record signals for the individual drops as fluorescence signal over a period of time, where one peak equals one drop (Fig S4 Insert).

Counting a given number of drops N_{drops} in a certain time span Δt allowed us to find the droplet frequency and calculate the exact droplet volumes.

Supplementary movies

Movie S1. Start of incubation (t= 0) for encapsulated trypanosomes in 0.2 nL droplets.
Movie S2. 24 hours of incubation for encapsulated trypanosomes in 0.2 nL droplets.
Movie S3. 48 hours of incubation for encapsulated trypanosomes in 0.2 nL droplets.
Movie S4. Start of incubation (t= 0) for encapsulated trypanosomes in 0.5 nL droplets.
Movie S5. 24 hours of incubation for encapsulated trypanosomes in 0.5 nL droplets.
Movie S6. 48 hours of incubation for encapsulated trypanosomes in 0.5 nL droplets.
Movie S7. Start of incubation (t= 0) for encapsulated trypanosomes in 2 nL droplets.
Movie S8. 24 hours of incubation for encapsulated trypanosomes in 2 nL droplets.
Movie S9. 48 hours of incubation for encapsulated trypanosomes in 2 nL droplets.

[1] D. C. Duffy, J. C. McDonald, O. J. A. Schueller, and G. M. Whitesides, Anal. Chem. **70**, 4974 (1998), ISSN 0003-2700, URL `https://doi.org/10.1021/ac980656z`.

[2] K. Eyer, R. C. L. Doineau, C. E. Castrillon, L. Briseño-Roa, V. Menrath, G. Mottet, P. England, A. Godina, E. Brient-Litzler, C. Nizak, et al., Nature Biotechnology **35**, 977 (2017), ISSN 1087-0156, 1546-1696, URL `http://www.nature.com/doifinder/10.1038/nbt.3964`.

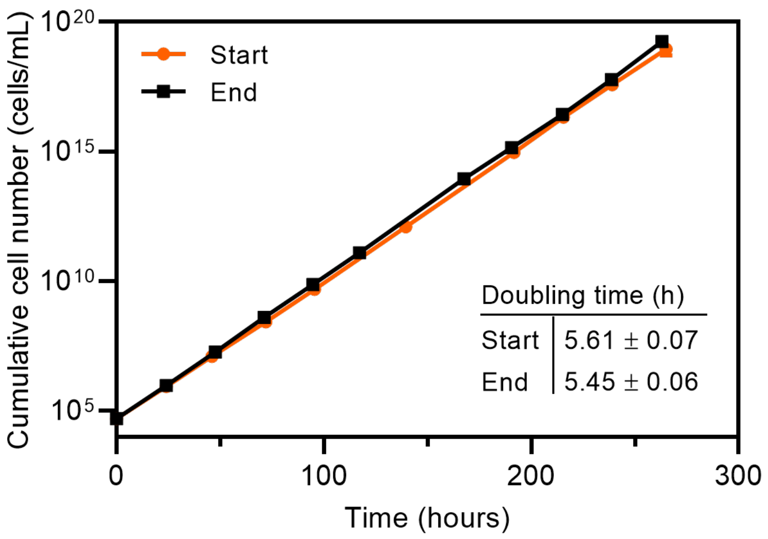

FIG. S1: Growth curves of *T.b. brucei* in bulk culture. Growth curves are presented as logarithmic accumulated cell number over time during a two-week period. Cells are counted at the beginning (orange) and the end (black) of the survival and growth experiments. Inserted table shows the respective doubling times from the start and the end measurements. All data are represented as mean values ±SD of technical replicates (n = 3).

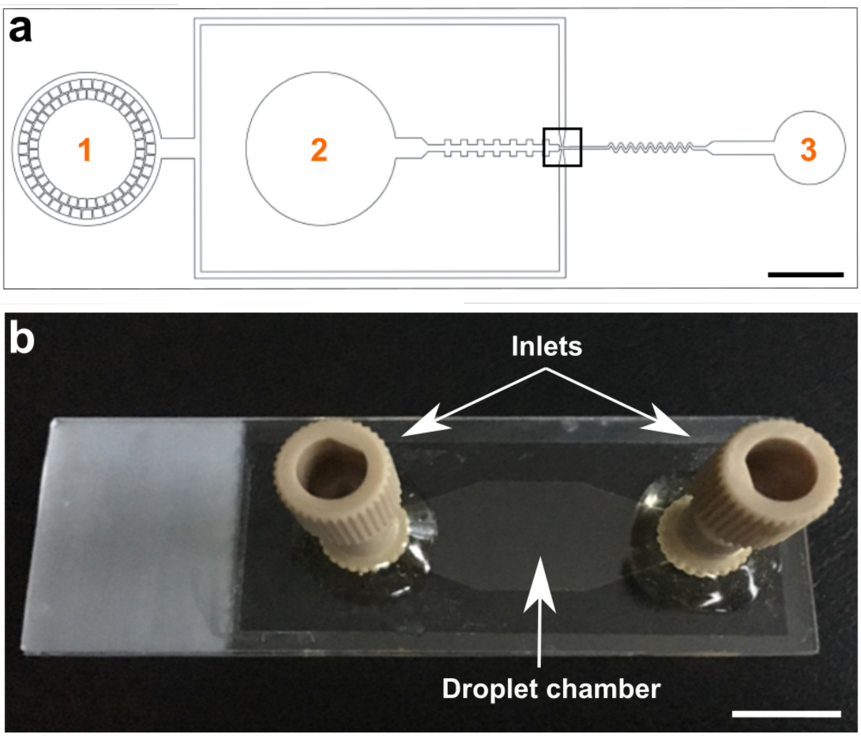

FIG. S2: Microfluidic devices for *T.b. brucei* encapsulation and incubation. (**a**) CAD design of a drop maker for single-cell encapsulation consisting of the oil inlet (1), the cell suspension inlet (2) and the collection outlet (3). The black square represents the microscopic image presented in Fig. 1b. Scale bar is 1 mm. (**b**) Incubation chamber enabling mono dispersed arrangement of drops adapted from Eyer *et al.* (2017)[2]. Scale bar is 12 mm.

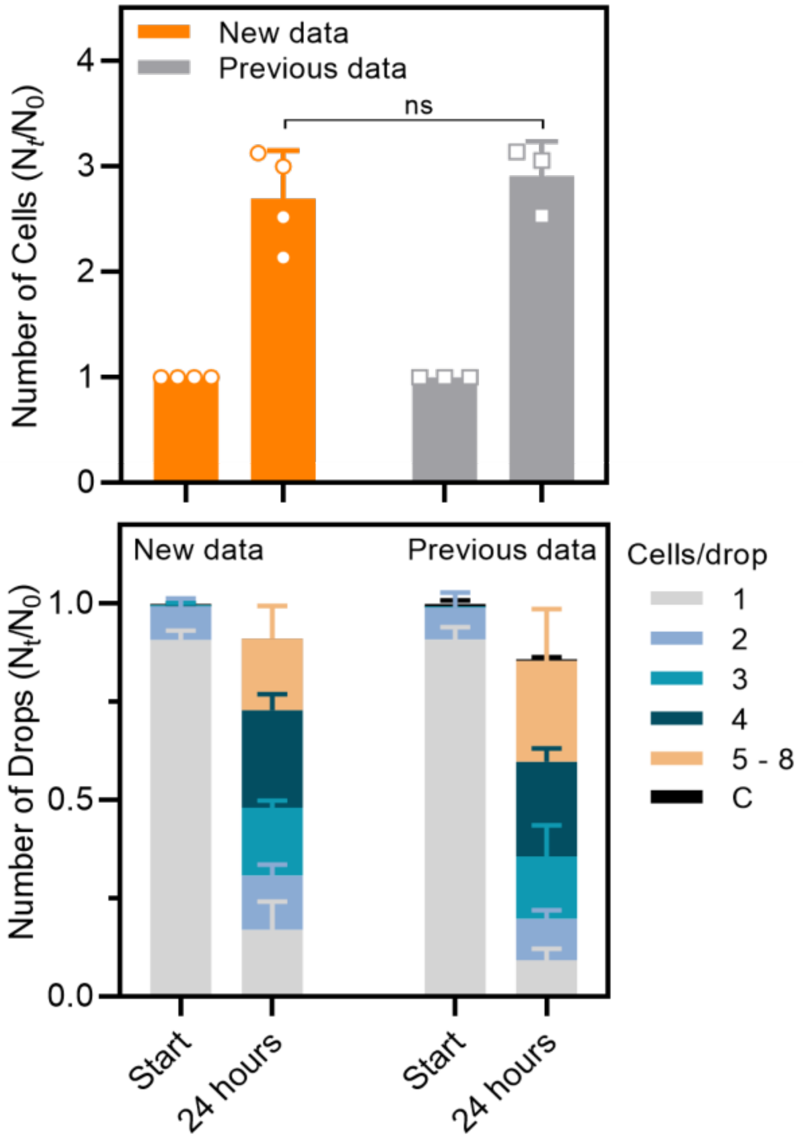

FIG. S3: Collection and incubation of *T.b. brucei* in 0.5 nL drops. Comparison of Previous data and New data of cell survival as normalized values after collection and 24 hours of incubation in two-dimensional incubation chamber (upper). Supporting cell survival data is the presentations of growth after 24 hours as normalized number of drops containing a given number of cells (lower). All data are presented as mean values ±SD. Two-sided Student's t-test were used in the upper graph to find significance value ($P = 0.53$) with $n = 3$ in Previous data and $n = 4$ in New data as biological replicates.

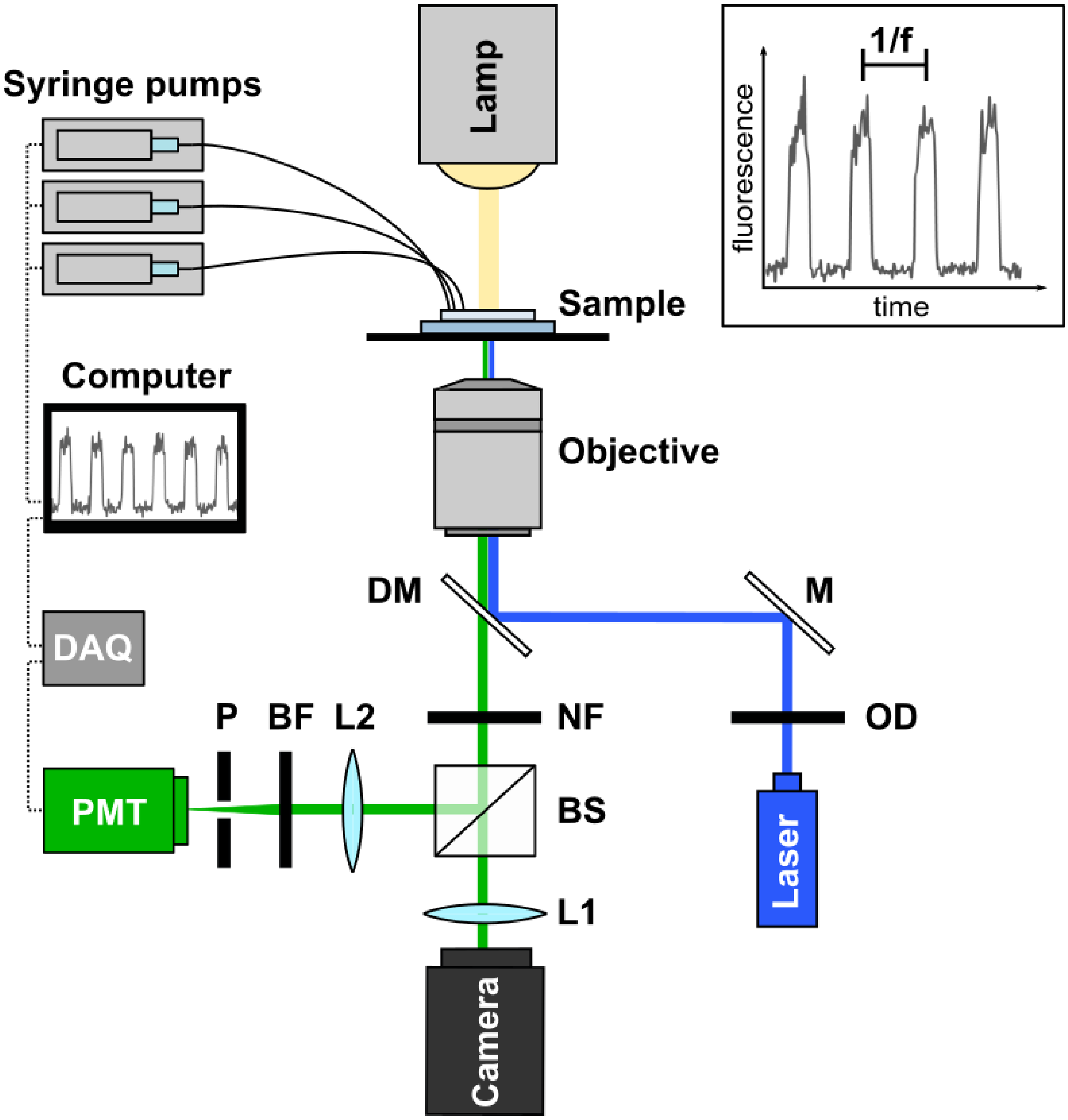

FIG. S4: Illustration of microfluidic setup used for all experiments. The setup is constructed by implementing the following components to an inverted microscope; syringe pumps, computer for control and acquisition, data acquisition (DAQ) card, photomultiplier tube (PMT), pinhole (P), band pass filter (BF), PMT lens (L2), dichroic mirror (DM), notch filter (NF), beam splitter (BM), camera lens directly from microscope (L1), mirror (M) and optical density (OD). Inset: Example of recorded signal from the drops used to calculate the droplet sizes. Illustration is created in Inkscape (https://inkscape.org).
